# 

**Published:** 1806-12

**Authors:** 


### NEW MEDICAL PUBLICATIONS.

The fourth edition, considerably enlarged and corrected, The Domestic Medical Guide, in Two Parts.—Part 1st. The Family Dispensatory, or Complete Companion to the Family Medicine Chest, with an Appendix, including the Management of Children; the Treatment of Poisons, suspended Animation, and such Cases of Emergency, which properly belong to Domestic Medicine, &c.—Part 2d. A new and complete System of Domestic Medicine; or familiar Treatise on the Prevention, Cure, and Distinction of Diseases, &c. &c. By Richard Reece, M. D.

An Essay on the Causes and Phenomenon of Animal Life. By John Herdman, M. D. 4s. boards.

Sketch of the Revolutions of Medical Science, and Views relating to its Reforms By P. T. G. Cabinis. Translated from the French, with Notes, by A. Henderson, M. D. 8vo. 9s. boards.

A System of Chemistry. By I. Murray. 4 vols. 8vo. 42s. boards.

### *TO CORRESPONDENTS.*

Communications are received from Dr. Bellamy, Dr. Hall, Mr. Harriss, Mr. Marson, Mr. Verral, Mr. Burns, and Mr. Hill.

END OF VOL. XVI.

### INDEX.

A.

ABDOMINAL mufcles, cafe of abfcess in the 77
Abernethy's Mr. furgical obfervations, reviewed 271
Abfcefs in the abdominal mufcles 77
Acacia, fubftitute for the Ægyptian 167
Acid fumigations, efficacy of 190
Acids, on the preparation of 103
——— their effects on calculi 225
Adams, Dr. on hydatids 97
———, on herpes in vaccination 247
———, on the fmall-pox 249
———, Mr. Young's letter to 403
Æther, its efficacy in cataract 2
Affufion cold, its effects in typhus 237
Aged patients, obfervations on 93
Ague, remedies for 167, 265
Albumen, obfervations on 302
Alcohol, its affinity with oxygen 533
Alkalies, experiments with 499
Alkaline fubftances, their effects on calculous concretions 221, 305
America, ftate of literature in 479
American difpenfatory, reviewed 570
Amputation, on the ligature of arteries after 79
——————— of the uterus, cafe of 472
Analogy, on reafoning from 115, 423
Anafarca pulmonum, on 354
Anderfon's Dr. cafe of teeth and hairs in the ovarium 80
Aneurifm, on fpurious 87
Angina pectoris, cafe of 487
Animal concretions, on 3
——— fubftances, on the putrefaction of 102
Animalcular exiftence of cancer on the 403
Animation, cafe of fuspended 529
Antimony, on the ufes of 428
Antifcorbutic, an excellent 264
Anti-vaccinarian fociety, account of the 476
——————— remarks on the 27
Aorta, on the offification of the 452
Apartments how to clean them from flies 190
Aphthous cafes, ufe of houfeleek in 76
Apoplexia hydrocephalica, on 117, 335
Apoplexy, obfervations on 530
Aqua kali puri, experiments with 502
Arabian horfes, on the greafe in 320
Areolar difpofition, on the 518
Arfenic, cafe of a perfon poifoned by 95
——— effects of the folution of 113
Arteries, on divided 87
——— on the ligature of 79
Afcites, cafe of encyfted 79
Afthma, on the humoral 354
Atkinfon, Mr. on the ufe of the feton 208

B.

Barker, Mr. on Arabian horfes 320
Bartholomew's hofpital, lectures at 285
Bathing recommended 281
Bath waters, experiments on the 383
——— obfervations on 434
Beddoes Dr. on vaccination 43
Beech, defcription and ufes of the 74
Belladonna, its ufe in inflammation of the iris 55
Bellamy's Dr. cafe of difeafed bladder 141
————— remarks on 251, 253
Biliary concretions, on 3
Bird's-cherry, botanical defcription of the 76
Blackberries, their ufe in gravel 263
Bladder, cafe of difeafed 141
————— remarks on 251, 253
Blair, Mr. on the vaccine conteft 475
Blood, converfion of chyle into 199
——— on letting of in yellow fever 413
——————— in fufpended animation 529
Body, on transformations in the 301, 451, 517
Bones, on their converfion into membranes 451
BOOKS, NEW MEDICAL, ANNOUNCED. Murray's Syftem of Chemiftry, 96.—General Index to the Medical Journal, *ib.* Sibthorp's Flora Græca, 192.—Cooper on Hernia, *ib.* Dr. Adams on Morbid Poifons, 288. Dr. Hamilton on Gout, 384. Mr. Young on the adhefive Strap, *ib.* Mr. Barclay on the Mufcles, 480. Mr. Burns on Hæmorrhage, *ib.* Dr. Cogan on the Paffions, *ib.* Mr. Vetch on Ophthalmia, *ib.* Sir John Sinclair's Code of Health and Longevity, 576
BOOKS, CRITICAL ANALYSIS OF NEW MEDICAL.—The Edinburgh Medical and Surgical Journal, 77, 172, 470.—Cow-pock Inoculation, vindicated by Rowland Hill, 82. Dr. Jones's Treatife on Hæmorrhage, 87. The Domeftic Medical Guide, by R. Reece, 180. Expofition on the Inoculation of the Small-pox, by J. C. Lettfom, M. D. 181. Encyclopœdia of Surgery, Medib cine, &c. by J. J. Wall, 182. An Examination into the Principles of the Brunonian Syftem, by Thomas Harrifon, *ib.* Dr. Pinckard's Notes on the Weft Indies, 268. Surgical Obfervations, by John Abernethy, 271. Remarks on the State of the Practice of Phyfic in Great Britain, by E. Harrifon, M. D. 279. Commentaries on the Lues Bovilla, by B. Mofeley, M. D. 280.—Medical Collections on the effects of Cold, as a remedy in certain Difeafes, by J. E. Stock, M. D. 366. Treatife on Vaccine Inoculation, by R. Willan, M. D. 372. The Vaccine Conteft, by W. Blair, 475. Remarks on Mr. Birch's Serious Reafons, by James Moore, 477. An Anfwer to Mr. Birch, by John Ring, *ib.* Practical Obfervations on the principal Difeafes of the Eyes, tranflated from the Italian of Anthonio Scarpa, by James Briggs, 562. Obfervations on Indigeftion, by M. Daubenton, 569. The Naval, Military, and Private Practitioner, by Ralph Cuming, M. D. 570. The American Difpenfatory, by John Redman Coxe, M. D. *ib.*
Botanical defcription of Britifh plants 73, 165, 261, 557
Bowels, on diforders of the 283, 381
Bozzini's Dr. light fpreader, defcribed 576
Bradley, Mr. on hernia 45
Brain, on offification of the 452
——, on tubercles in the 470
Breweries, their effects on health 101
Bronchocele, on 207
Brunonian fyftem, on the 183
Buchan, Dr. on drinking cold liquors 383
Buchner, Mr. on inoculation 478
Burnet-rofe, defcription of the 261
Burns, houfeleek a remedy for 76
——, on the cooling treatment of 8, 210, 526
——, on ol. terebinth. in 313
Burns, Mr. on crural hernia 172
——— obfervations on 119

C.

Cairo, drunkennefs of the inhabitants of Grand 164
Calculous concretions, experiments on 153, 221, 305, 497
Calculus, account of a urinary 3
Cambridge, on taking medical degrees at 344
Camel, on the anatomy of the 191
Cancer, obfervations on 97, 403
——— effects of diæta aqua on 383
Cancerous hydatids, on 97, 403
Cantharides, on the internal ufe of tincture of 78
Canton, vaccination at 576
Carlfbad, account of the waters of 504
Catalepfy, cafe of 525
Cataract, on the caufes of 2
Cauftic, its ufe in hydrophobia 440
Cayley, Dr. on medical reform 347
Celandine, botanical defcription of 558
Cellulo-calcareous difpofition, on the 520
Cerebrum, excrescence from the 149
Chalmers, Mr. on difeafed bladder 251
————, on pulmonic inflammation 481
Champagne wine, properties of 162
Charcoal, effects of the fumes of 369
Chemical fociety, inftitution of a 96
Cherry-tree, on the gum of the 166
Chefnut, defcription of the 73
———— longevity of the 74
China, vaccine inoculation in 96
Cholera, on the treatment of 381
Chyle, its converfion into blood 199
Cinnabar, component parts of native 575
Cinque foil, defcription of the 265
Clark, Mr. on the ftone 554
Clarke's Dr. cafe of difficult parturition 53
——— cafe of amputation of the uterus 472
Clement, Mr. on vaccination 69
———— anfwer to 67
Cloud-berry, botanical defcription of the 263
Cold, its effects in certain difeafes 366
————— in burns 17
Collins's Mr. cafe of retention of urine 250
Concretions, on animal and biliary 3
————, on inteftinal 4
————, on gravelly and calculous, 153, 221, 305, 497
Conferve of hepps, compofition of 262
Confumption, on digitalis in 229, 353
Contagion, a prefervative againft 81
————, on preventing 190
Controverfy, on intemperate 247
Convulfions, ufe of emetics in 432
Coote's Mr. cafe of epilepfy 473
Corns, a remedy for 75
Correfpondents, to 96, 192, 288, 384, 480, 576
Coup de foleil, cafe of 269
Cow-pock matter, on the origin of 328, 441
Cow-pox, on a claffical title for the 334
————, on fuppofed failure of 460
Coxe, Dr. on the treatment of burns 9
Crab-tree, defcription and ufes of the 170
Creafer, Mr. on vaccination 24
——— anfwer to 65
Croup, ufe of emetics in 430
Crowther's Dr. cafe of abfcefs 77
Cumming's Dr. naval, military and private practitioner, reviewed 570
Cyder, account of the different kinds of 170
Cynanche maligna, on the treatment of 430

D.

Dancer's Dr. reply to Dr. Grant 410
Davies, Mr. on rheumatifm 495
Davis, Mr. on rabies canina 437
Davy's Mr. account of a new foffil 191
Dawfon, Mr. on difeafed bladder 253
Deafnefs, effects of galvanifm in 472
Defour's Mr. cafe of plica polonica 235
Degrees, on conferring medical 344
Delivery, cafe of premature 4
Denmark's Mr. cafe of gaftritis 535
Diarrhœa, virtues of the tormentil in 266
——— on the treatment of 283
——— cafes of 467
Digitalis, its efficacy in confumption 229, 353
———, on the ufes of 424
Difeafes, account of in an eaftern diftrict of London, from May 20, to June 20 93
——— from June 20, to July 20 187
——— July 20, to Aug. 20 283
——— Sept. 20, to Oct. 20 466
——— Dr. Fothergill's report of 90, 185, 280, 380, 467, 571
——— at Lancafter, report of 188
——— effects of cold on certain 366
Dog's-rofe, botanical defcription of the 261
Drake Dr. on difeafed fpleen 471
Dropfy, ufe of oil in 81
Drop-wort, botanical defcription of 171
Druggifts, on the practices of 458
Dumas, M. on changes in the human body 301, 451, 517
Dunning, Mr. on vaccination 574
Dunning's Mr. letter to Dr. Clarke 260
Duodenum, on diftention of the 302
Dutch-pink, how extracted 75
Dyfenteries, houfeleek good in 76
——— virtues of the tormentil in 267
——— ufe of emetics in 432

E.

Earth, on the internal temperature of the 192
Edinburgh Medical Journal, reviewed 77, 172, 470
——— cuftom at taking medical degrees in 456
Edward's Mr. cafe of fcurvy 113
Egan, Dr. on gravelly and calculous concretions 153, 221, 305, 497
Egremont, Lord, on vaccination 36
Egyptian ophthalmia, remedy for 283
Electricity, important fact in 190
Elephantiafis, cafe of 363
Emetics, on the ufes of 424
Epilepfy, ufe of emetics in 432
———, cured by trepanning 473
Equine difeafe, fame as cow-pox 125, 239
Eruptions, on herpetic 71
Eryfipelas, ufe of emetics in 432
Euphorbia, botanical defcription of 75
Evans, Dr. on the cooling treatment of burns 461, 527
Excitement, on the word 184
Experiments on arfenic 95
——— on teeth and ivory ib.
——— vaccine 123, 240
——— on gravelly and calculous concretions 153, 221, 305, 497
——— in electricity 190
——— on the temperature of the earth 192
——— on the Bath waters 383, 434
——— Galvanic 479
Eyes, on difeafes of the 55, 208, 562

F.

Femoral artery, on compreffing the 14
Fermor, Mr. character of 539
——— letters of 541
Fever, report on contagious 288
Fevers, ufes of emetics in 432
Fibrous difpofition, on the 519
Fiftula in perinæo, cafe of 141
Flax, on the fteeping of 101
Flies, how to clear rooms from 190
Flora græca, account of the 192
Fluoric acid in teeth and ivory 95
——— in a new foffil 191
Fœtuffes, on extra uterine 385
Foffil, analyfis of a new 191
Fothergill's, Dr. report of difeafes 90, 185, 280, 380, 467, 571
Fracture, cafe of, fungous after 149
Friend, Dr. on the treatment of fmall-pox 63
Fumigations, on anti-contagious 190
Fungous, cafe of after fracture 149

G.

Gall bladder, on the ufes of the 201
Galvanic fluid, its effect on vegetable infufions 479
Galvanifm, its effects in deafnefs 472
Gardner's, Mr. Galvanic experiments 479
Gaftritis, cafe of 535
Gelatine, on 304
Geum, botanical defcription of 267
Gibbes, Dr. on the Bath waters 383, 434
Glafgow, medical lectures in the univerfity of 286
Gleet, ufe of tincture of cantharides in 78
Gonorrhœa, horfe-leek good for 76
Goodall, Mr. on burns and fcalds 313
Good Hope, vaccine inoculation at 42
Gout, ftrawberries good for the 264
———, on lime water in 312
Grant, Dr. on yellow fever, remarks on 410
Granular difpofition, on the 520
Gravel, efficacy of blackberries in 263
Gravelly concretions, on 153, 221, 305, 497
Greafe in horfes, on the 124, 215, 321
———, its ufe among the Hottentots 8[illegible]

Groin, on compressing the femoral artery at the 14

Guinea worm, remarks on the 79

———, history of the 177

Gum, virtues of the cherry tree 166

Guy's hospital, lectures at 285

Gwennap mines, new fossil found in the 95

H.

Hæmoptysis, on the cure of 431

Hæmorrhage, observations on 87

———, on secondary 79

——— from the nose, how to stop 191

———, on the causes of 196

Hamilton, Dr. on digitalis 229

Hanging, case of suspended animation by 529

Harrison, Dr. on medical reform 279

Harrold, Mr. in answer to Dr. Kinglake 115

Harvey's discovery of the circulation of the blood 31

Head ache, remarks on 372

Health, on manufactures prejudicial to 100

Hemiplegia, case of 187

Hepatic bile, on the 200

Hepps, how to make conserve of 262

Herb fennel, virtues of the 267

Hernia, on the treatment of 45

———, cases of 48

———, case of crural 81

———, observations on 119

———, case of sphacelated 178

———, on an adherent omental 273

Herpes, observations on 178

——— in vaccination, on 247

Hill's, Mr. cases of inoculaation 110

——— R treatise on vaccination, reviewed 82

Hindoos, their method of curing the Guinea worm 177

Hippocrates, on the virtues of oil 80

Home, Mr. on the anatomy of the camel 191

Hooping cough, on nitrat of silver in 16

———, use of emetics in 431

Horses, on the grease in 124, 320

———, how to keep flies from 190

Hottentots, use of grease among the 80

House-leek, description and virtues of the 75

Humboldt on the physiognomy of plants 95

Humerus, change in the articulation of the 518

Hunter, Mr. on the external use of oil 80

Hydatid, existence of the cancerous 97, 403

Hydrophobia, on the virus of 439

Hydrothorax, case of, with remarks 229, 353

I.

Idiopathic tetanus, cases of 473

Indigestion, on 569

Inflammation, on pulmonic 481

———, case of peritoneal 513

Inoculation, cases of 110

Insanity, use of emetics in 432

Intelligence, medical and physical 94, 188, 283, 383, 478, 573

Intermittents, remedy for 559

Intestinal concretions, on 4

——— canal, on the term 291

Iris, on division of the 1

———, on inflammation of the 55

Itch, remedy for 559

Ivory, fluoric acid in 95

J.

Jenner, Mr. H. on vaccination 33

———, Dr. on vaccination 35

———, letter to 37

Jennerian institution, proceedings of the royal 478

Johnson's, Mr. supposed case of small-pox 473

Jones, Mr. on hooping cough 16

———, Dr. on hæmorrhage 87

K.

Kali preparatum, its effects in scarlatina 553

Kelly's, Mr. case of sphacelated hernia 178

Kentish, Dr. on burns and scalds 8

Key, Mr. answer to 550

——— on supposed cow-pock failure 460

Kidd, Dr. on a new metal 95

King's, Mr. case of elephantiasis 363

Kinglake, Dr. on the treatment of burns 17, 480, 526

——— in reply to Dr. Kentish 210

——— in answer to Mr. Ring 328

Kite, Mr. on cataract 1

Kitson's, Mr. case of tic doloreux 178

Klaproth, M. on cinnabar 575

Knowles, Dr. on vaccination 332

———, appointed physician to the Jennerian society 478

Koutam-poulli, on the 79

L.

Lafont's, Dr. vaccine experiments 241
Lancaster, report of diseases in the dispensary at 188
Laurence, Mr. on vaccination 214
LECTURES announced:—At St. Thomas's Hospital, Mr Cline and Mr. Cooper on Anatomy and Surgery, 285. Guy's Hospital, Drs. Babington and Curry on the Practice of Medicine, Dr. Babington and Mr. Allen on Chemistry, Doctor Curry on the Theory of Medicine, Dr. Haighton on Physiology and Midwifery, Drs. Babbington, Curry, and Marcet's Clinical Lectures, Mr. Fox on the Teeth, Mr. Coleman on Veterinary Medicine, 285. At the London Hospital, Dr. Cooke on the Theory and Practice of Phyfic, Drs. Hamilton and Yellowly on Chemiftry, Dr. Frampton on Materia Medica, Dr. Dennifon on Midwifery, Meffrs. Blizard on Surgical Cafes, Meffrs. Headington and Frampton on Anatomy, Phyfiology, and Surgery, Mr. Armiger's Anatomical Demonftrations and Diffections, Mr. Headington on Surgery, 285. At the Medical Theatre, St. Bartholemew's Hofpital, Drs. Roberts and Powell on the Theory and Practice of Medicine, Mr. Abernethy on Anatomy, Phyfiology, and Surgery, Mr. Macartney on Comparative Anatomy and the laws of Organic Exiftence, Dr. Edwards on Chemiftry, Dr. Thynne on Midwifery and the Difeafes of Women and Children, Mr. Lawrence's Anatomical Demonftrations and Practical Anatomy, 286. Univerfity of Glafgow, Dr. Miller on Dietetics, Materia Medica, and Pharmacy. Mr. Towers on Midwifery, Dr. Freer on the Theory and Practice of Phyfic, Dr. Jeffray on Anatomy and Surgery, Dr. Cleghorn on Chemiftry and Chemical Pharmacy, Dr. Freer's Clinical Lectures, Dr. Brown on Botany, 286. Mr Wilfon on Anatomy, Phyfiology, and Surgery, 286. Mr. Brookes on ditto, *ib.* Dr Badham on Medicine and Chemiftry, *ib.* Dr. Bradley on the Theory and Practice of Medicine, *ib.* Mr. C[illegible]rlifle on Surgery, 287. Dr. Clarke and Mr. Clarke on Midwifery and the Difeafes of Children, *ib.* Dr. Pearfon on the Practice of Phyfic, Therapeutics, and Chemiftry, *ib.* Dr Reid on the Theory and Practice of Medicine, *ib.* 384. Mr. Home on Surgery, 287. Mr. Gunning on ditto, *ib.* Mr. Thomas on ditto, *ib.* Mr. Moor on the Structure and Difeafes of the Teeth, 288, 384. Mr. Thelwall on Elocution, 288. Drs. Dennifon and Squire on Midwifery and the Difeafes of Women and Children, 383. Mr. Gardner on Chemiftry, 384. Dr. Hooper on the Theory and Practice of Phyfic, *ib.* Mr. Milburne on Anatomy, Phyfiology, and Surgery, 480. Dr. Clough on Midwifery, 576. Mr. Alcock on Anatomy and Surgery, *ib.*

Leefe's, Mr. cafe of ftricture of the urethra 108

Legs, cafes of difeafed 362

Lettfom, Dr. on variolous inoculation 35

——— expofition on inoculation 181

Leucorrhœa, ufe of cantharides in 78

Ligature, on the operation of the 88

Light fpreader, a new inftrument defcribed 576

Lime water, experiments on 497

Lithotomy, on the operation of 162

Liver, on hydatids in the 97

——, on the functions of the 198, 294

——, on fchirrhofities of the 433

London, monthly report of difeafes in 90, 185, 280, 380, 466, 571

——— hofpital, lectures at 285

Loy's, Dr. vaccine experiments 123, 240

Lungs, inftance of a change of the 304

———, ufe of emetics in certain affections of the 431

M.

Macleay's, Dr. cafe of afcites 79

Macnamara, Capt. on vaccination 188

Mammothis, cafe of 365

Manchefter, report of the board of health at 338

———, practice of druggifts at 458

Manufactures prejudicial to health 100, 176

Marfon, Mr. on vaccination 70

Meafles, remedy for the 171

Medical degrees, on taking 344

——— reform, on 94, 345, 347, 349, 453

——— and phyfical intelligence 94, 188, 283, 383, 478, 573

——— publications, lift of new 192, 288, 384, 480, 576

Medlar-tree, botanical defcription of the 169

Medullary difpofition, on the 518

Mental pathology, on the ftudy of 474

Mercury, on its tendency to produce putrefcency 415

Merriman, Mr. on retroverfion of the womb 385

Mind, its influence on the animal functions 92

Mineral, account of a new 95

Mines, on heat in 192

Mogols, vaccination among the 192

Moore, Mr. on the anti-vaccinifts 29

——— his anfwer to Mr. Birch 477

Mofeley, Dr. on the cow-pox, reviewed 280

Muriatic acid, on the preparation of 104

Murfinna, Mr. on tetanus 473

Mufcles, on the changes of 303

———, on the offification of the 452

N.

National inftitute, report of the French 100, 176

Neyle, Mr. on inoculation 138

Nitrat of filver, virtues of 16

Nitre, its ufe in fphacelus 114

Nitrous acid, preparation of 104

Nofe, cafe of polypus of the 7

——, how to ftop hæmorrhages of the 191

Nottingham, report of vaccination at 132

O.

Odier's Dr. account of the death of M. de Sauffure 470

Oefophagus, on fpafmodic ftrictures of the 277

Oil, on the external ufes of 80
—— of beech, ufed for butter 74
Okes, Mr. on the ophthalmia 575
O'Leary, Dr. on yellow fever 490
Ol. terebinth. in burns, efficacy of 313
Omental hernia, cafe of 273
Ophthalmia, remedies for the 283, 575
———— obfervations on 567
Opiates in diarrhœa, effects of 467
Opium, its ufe in pneumonia 486
————, its efficacy in rheumatifm 496
Organs, on the white induration of 470
————, on the ftructure of human 518
Offification of the brain, on the 452
Otaheite, on the depopulation of 174
Ovarium, hair and teeth in the right 80
Oxygen gas, its benefit in reftoring fufpended animation 533

P.

Pancreas, on the functions of the 201, 298
Paralyfis, obfervations on 187
Paraphymofis, cafes of 140
Parturition, cafe of unufual 53
Pathology, on the ftudy of mental 474
Patton, Mr. on the guinea worm 79
Pear-tree, defcription and ufes of the 170
Percival, Dr. on the practice of phyfic 459
Peripneumony, cafe of 468
Peritonæal inflammation, cafes of 513
Perkins's tractors, quackery of 9, 457
Perry, qualities of 170
Pfaff, Prof. on a cafe of poifon 95
Phlegmafia, ufe of cold in the 371
Phyfic, on the prefent ftate of 279
———— plan for regulating the practice of 345
Pinckard, Dr. on the Weft Indies, reviewed 268
Placenta, on the burning of the 54, *note.*
Plague, oil a prefervative agafnft the 81
Plants, on the phyfiognomy of 95
———— botanical defcription of Britifh 73, 165, 261, 557
Pleurify, cafe of 469
Plica polonica, cafe of 235
Plum-tree, defcription of 166
Plymouth, proceedings of the Jennerian Society, at 43
Pneumonia, predifpofing caufe of 483
Poifon, cafe of 95
Poland, progrefs of vaccination in 41
Polypus of the nofe, cafe of 7
Poor, report of fociety for bettering the condition of the 288
Poppy, defcription and virtues of the 560
Potentilla, defcription and ufes of 264
Poudrette, on the manufacture of 102
Premature delivery, cafe of 5
Pruffian blue, on the manufacture of 105
Pulmonary organs, on the change of the 304
Pulmonic inflammation, on 481
Pulfe, effects of cold on the 367

R.

Rabies canina, on 437
Rafpberry, defcription and ufes of the 262
Retroverfion of the womb, cafes of 386
Reyfs's Dr. letter to Dr. Jenner 37
Rheumatifm, on the treatment of 495
Ring, Mr. on compreffing the femoral artery 14
———, on vaccination 23, 239, 313
———, in anfwer to Dr. Kinglake 122, 441
———, in reply to Dr. Walker 317
———, on cutting up a vaccine veficle 323
———, his anfwer to Mr. Birch 477
———, in reply to Mr. Southams 539
———, letters from Mr. Fermor to 541
Ring-worms, remedy for 559
Roberton, Mr. on cantharides 78
Rofa, botanical defcription and virtues of 261
Ruptures, remedy for 171
Rufh, Dr. on the fpleen, &c. 193
———— anfwer to 289

S.

Salts, on earthly calcareous 452
Saumarez, Mr. on the functions of the liver 289
Saunders, Mr. on inflammation of the iris 55
Sauffure, M. de, account of the death of 470
Scalds, on the treatment of 8, 17, 210, 319, 526
Scarlatina anginofa, cafes of 94
———— anginofa, obfervations on 552
Scurvy, cafe of inveterate 113
———— remedy for the 267
Seton, on the ufe of the 208
Shawe, Mr. on ophthalmia 283
Shearly's Mr. cafe of peritoneal inflammation 513
Sibthorpe's Flora Græca, account of 192
Simmons's Mr. cafes in furgery 1
Simpfon's hofpital in Dublin, account of 510
Skin, cafe of fyphilitic ulceration of the 77
Slaughter houfes, obfervations on 102
Sloe-tree, defcription and ufes of the 168
Small-pox, ravages of the 23
————, on confluent 62
————, Dr. Friend on the treatment of 63
————, cafe of vaccination after 68
————, remedy for expelling of 17[illegible]
———— inoculation, on 139
————, Dr. Adams on the 24[illegible]
————, cafe of fuppofed 473
————, on inoculation for the 478
Society iflands, ftate of the 176
Soda water, experiments on 49[illegible]

Sorbus, botanical defcription of the 169
Southams, Mr. on vaccine inoculation 436
————, reply to 539
Spalding, Mr. anecdote of 533
Sphacelated hernia, cafe of 178
Sphacelus, ufe of nitre in 114
Spleen, on the ftructure and functions of the 194, 300
————, cafe of difeafed 471
Sprains, remedy for recent 170
Spurge, ufes of the red 75
Staunton, Sir G. tranflates a treatife on vaccination into Chinefe 96
———————— on vaccination at Canton 573
Stock, Dr. on cold as a remedy in certain difeafes 366
————, on the medical effects of cold water 465
Stomach, on difeafes of the 422
Stone, cafes of the 555
————, on the caufes of the 162
Stone's Dr. treatife on the ftomach, obfervations on 422
Strawberry, botanical defcription and virtues of the 264
Suffolk medical fociety, refolutions of the 94
Sully, Mr. on the ravages of the fmall-pox 23
Sulphuric acid, preparation of 103
Surgery, felect cafes in 1
Surgical obfervations 271
Sutton's practice, on 72
Switzerland, on the difeafes of 163
Synochus, obfervations on 186
Syphilitic ulceration of the fkin 77

T.

Taxis, on reduction by the 45
Tea leaves of the floe a fubftitute for 168
Teeth, fluoric acid in human 95
————, how to diffolve the tartarous concretions of the 263
————, a gargle for loofe 265
Tetanus, cafes of idiopathic 473
Tetters, remedy for 559
Thackeray, Mr. on the treatment of 344
Thomas's, St. hofpital, lectures at 285
Thornton, Dr. on fufpended animation 529
Thrufh in children, remedy for 76
Thyroid gland, on the functions of the 207
Tibia, caries of the 77
Tic doloreux, cafe of 178
Tinct. ferri muriati, effects of 250
Tooth-ache, remedy for the 75
Tormentil, defcription and virtues of the 265
Transformation of fubftances, obfervations on 303, 451, 517
Trepanning, epilepfy cured by 473
Trichiafis, on 564
Tubercles in the brain, on 479
Turpentine, its efficacy in burns 10, 116, 313
Typhus, cafes of 236
————, recurrence of 382

U.

Ulceration, cafe of fyphilitic 77
Ulcers, ufe of houfe-leek in hot 76
Urethra, ftricture of the 108
Urinary concretions, on 3
Urine, cafes of incontinence of 554
————, cafes of retention of 140, 250
————, experiments on 225, 312
————, on variations of the 274
Uterus, on wounds of the 402
————, cafe of amputation of the 472

V.

Vaccination, Mr. Sully on 23
————, Mr. Creafer on 24
————, Mr. Ring on 23, 239, 313, 441, 539
————, Dr. Reyfs on 37
————, Dr. Beddoes on 43
————, teftimonial in favour of 44
————, Dr. Wood on 68
————, Mr. Marfon on 70
————, cafe of 72
————, in China 96, 578
————, Mr. Hill's cafes of 110
————, Mr. Neyle on 138
————, Capt. Macnamara on 188
————, Mr. Laurence on 214
————, Dr. Adams on 247
————, Mr. Dunning on 260, 574
————, report on 284
————, Dr. Knowles on 332
————, Dr. Willan on 372
————, Mr. Southams on 436
————, Mr. Key on 460
————, Dr. Wall, on 546
Vaccine inftitution, refolutions of the 284
————————, inftructions of the 325
Vaccine inoculation at Nottingham, report of 132
———— conteft, account of 475
———— veficle, danger of cutting a 323
Variolous eruptions, on 377
Vegetable infufions, effects of Galvanic fluid on 479
Venereal difeafe, obfervations on 172
————————, remedy for the 76
———————— ophthalmia, on the 567
Veficle, on the danger of cutting up a vaccine 323
————, on the irregular 375
Vinegar, on the diftillation of 104
Vifcera, on the changes of the 454
Volta, Prof. on Galvanifm 472

W.

Waldon, Dr. on fcarlatina 552

Wall, Dr. on vaccination 546

Wardrop's, Mr. case of hernia 81

Warren's, Mr. case of fracture 149

Warts, remedy for 75

Water, effects of cold, in burns 17, 526

———, in typhus 236

———, in cancer 383

———, on the medical effects of 465

———, in yellow fever, on 491

Wavellite, a new fossil, described 191

Weaver, Mr. on apoplexia hydrocephalica, answer to 117

——————— his reply 335

Weldon's, Mr. case of angina pectoris 487

Westminster dispensary, reports of diseases at the 91, 185, 280, 380, 467, 571

White, Mr. on the koutam-poulli 79

Wild woad, description and uses of 74

Willan, Dr. on vaccine inoculation 372, 448

Williams, Mr. on vaccination 28

Wilson, Mr. on the venereal disease 172

——————— on the depopulation of Otaheite 174

Womb, on retroversion of the 386

Wood, Dr. on confluent small-pox 62

Worm medicines, danger of 469

Y.

Yellow dye, description of a 75, 170

——— fever, cases of 268

———————, remarks on Dr. Grant's treatise on 410

———————, virtues of cold water in 4[illegible]0

York, duke of, his testimony in favour of vaccination 35

Young, Mr. on hydatids in cancer 403

---

### REFERENCES TO THE PLATES.

Mr. Simmons's Case of Intestinal Concretions - - - - 3
——————— Case of Nasal Polypus - - - - - *Ibid.*
——————— Cases of Urinary and Biliary Calculi - *Ibid.*
Mr. King's Cases of Diseased Legs - - - - - - - 362

